# Endothelial senescence of the vertebral artery in the context of cervical degeneration: biomechanically driven remodeling of the hemodynamic environment

**DOI:** 10.3389/fcell.2026.1839226

**Published:** 2026-05-21

**Authors:** Xiaomin Wang, Xiaoyu Liu, Luhao Yu, Yongpeng Xue, Kangyi Hu, Yongjia Song, Min Song

**Affiliations:** Clinical College of Traditional Chinese Medicine, Gansu University of Chinese Medicine, Lanzhou, China

**Keywords:** cellular senescence, cervical degeneration, endothelial cells, posterior circulation, vertebral artery

## Abstract

Cervical spine degeneration is frequently accompanied by long-term alterations in the hemodynamic environment of the vertebral arteries. Traditionally, vascular abnormalities associated with cervical degeneration have been interpreted mainly from the perspectives of structural compression or macroscopic hemodynamic disturbance. However, these explanations do not fully account for the functional instability of posterior circulation perfusion that may occur even in the absence of overt arterial stenosis. Emerging evidence suggests that endothelial senescence represents a central biological process linking chronic mechanical stress, disturbed flow patterns, and progressive vascular dysfunction. In the vertebral artery, which is anatomically coupled to the cervical spine and exposed to dynamic biomechanical forces, long-term alterations in vascular geometry and shear stress may gradually reshape the local microenvironment of the vascular wall. These changes can promote endothelial stress responses, metabolic imbalance, and inflammatory signaling, ultimately facilitating the development of endothelial senescence.In this review, we integrate current knowledge from vascular biology, mechanobiology, and cerebrovascular physiology to examine how cervical degenerative changes may remodel the vertebral artery microenvironment. We discuss the molecular basis of endothelial senescence, the role of disturbed flow in activating pro-senescent signaling pathways, and the potential consequences of endothelial aging for the functional stability of the posterior circulation. By framing vertebral artery–related abnormalities within the context of endothelial senescence and vascular microenvironment remodeling, this perspective provides a conceptual framework that may help explain chronic posterior circulation vulnerability associated with cervical degeneration and identify potential directions for future mechanistic and translational research.

## 1 Introduction

With the ongoing acceleration of global population aging, vascular aging has increasingly been recognized as a fundamental biological basis underlying a wide range of chronic diseases and functional decline. In this context, the vascular endothelium plays a central role in maintaining vascular homeostasis, regulating blood flow distribution, and preserving tissue perfusion. The functional state of endothelial cells therefore critically influences the overall performance and adaptability of the vascular system (Ahmed et al., 2024; Jia et al., 2019).

Recent studies have increasingly recognized that endothelial senescence is not merely a consequence of passive cellular deterioration or injury, but rather an active pathological process driven by multiple factors, including mechanical stress, metabolic burden, and chronic inflammatory signaling (Augustin and Koh, 2024). Senescent endothelial cells are typically characterized by permanent cell-cycle arrest, activation of the senescence-associated secretory phenotype (SASP), and systemic alterations in vascular functions such as vasomotor regulation, barrier integrity, and anti-inflammatory capacity. These changes collectively contribute to local dysregulation of blood flow control and the development of chronic hypoperfusion states (Brandes et al., 2005; Cheng C. K. et al., 2025; Pantsulaia et al., 2016). In the cerebrovascular system in particular, neural tissue exhibits a high sensitivity to alterations in perfusion, meaning that endothelial senescence–related vascular dysfunction may lead to disproportionately amplified physiological and clinical consequences (Bkaily and Jacques, 2023; Behringer, 2023).

The vertebral artery constitutes a key component of the posterior cerebral circulation and possesses anatomical and biomechanical characteristics that differ substantially from those of other large arteries. Running along the cervical spine, the vertebral artery maintains a close anatomical relationship with the vertebral bodies, intervertebral discs, and surrounding soft tissues. During physiological neck movements such as flexion, extension, and rotation, the vessel is continuously exposed to dynamically changing mechanical environments, including tensile forces, compressive stress, and complex patterns of hemodynamic shear stress (Magklara et al., 2021; Mitchell, 2005).

This anatomical mechanical coupling, which is highly dependent on the structural integrity and mobility of the cervical spine, renders the vertebral artery particularly susceptible to the combined influences of biomechanical and hemodynamic factors under both physiological and pathological conditions. Previous studies have demonstrated that abnormal shear stress patterns and disturbed flow are major stimuli for endothelial dysfunction. However, the potential significance of these mechanisms within the vertebral artery, an anatomically unique vascular bed has not yet received sufficient attention.

Existing research has largely focused on imaging-based structural abnormalities or macroscopic hemodynamic parameters when investigating vertebral artery alterations associated with chronic cervical structural and mechanical changes. These studies have explored phenomena such as restricted vascular course, luminal narrowing, and changes in blood flow velocity. While such findings have provided important insights into the relationship between cervical structural alterations and vertebral artery hemodynamic abnormalities, their explanatory framework remains largely confined to the “structure-flow” paradigm.

Consequently, relatively little attention has been directed toward biological remodeling within the vascular wall, itself particularly the responses of the vascular endothelium under conditions of long-term mechanical and metabolic stress. This limitation leaves several clinical and physiological observations insufficiently explained. For instance, patients may exhibit persistent or recurrent symptoms of posterior circulation hypoperfusion even in the absence of overt vascular stenosis or acute occlusion, suggesting that mechanisms beyond macroscopic structural abnormalities may be involved. These mechanisms may operate at a more subtle yet persistent microvascular or cellular leve (Hardin et al., 1960).

A growing body of evidence from vascular mechanobiology indicates that chronic exposure to disturbed flow, oscillatory shear stress, and metabolic stress can promote endothelial dysfunction and may induce endothelial senescence. These processes involve oxidative stress, mitochondrial dysfunction, DNA damage responses, and persistent inflammatory signaling. Within this context, endothelial senescence may act as an integrative biological interface through which mechanical and metabolic disturbances are translated into long-term vascular dysfunction. However, it should be noted that most of this evidence has been derived from studies in other vascular beds, such as the aorta, carotid artery, or coronary circulation, and its direct applicability to the vertebral artery remains uncertain.

Importantly, a critical limitation in the current field is the lack of direct evidence linking cervical degenerative changes to endothelial senescence specifically within the vertebral artery. To date, no studies have directly demonstrated endothelial senescence markers in human vertebral arteries in association with cervical degeneration, nor have observational studies established a clear causal relationship between these processes. Therefore, any proposed link between cervical degeneration and endothelial senescence should be interpreted with caution. Rather than representing a confirmed causal pathway, the conceptual framework presented in this review is intended as a hypothesis-driven integration of multiple lines of indirect evidence. First, extensive experimental studies have established that abnormal hemodynamic forces can induce endothelial senescence in various vascular systems. Second, anatomical and biomechanical studies suggest that cervical degeneration alters vertebral artery geometry and flow conditions. Third, clinical observations indicate that posterior circulation dysfunction may occur even in the absence of overt structural vascular abnormalities. Taken together, these observations support a biologically plausible, though as yet unproven, connection between cervical degeneration, altered hemodynamic environments, and endothelial aging.

Based on this perspective, the present review aims to synthesize current knowledge from vascular biology, mechanobiology, and cerebrovascular physiology to explore how cervical degenerative changes may influence the vertebral artery microenvironment. By framing these interactions within the context of endothelial senescence, we seek to provide a conceptual model that may help explain the functional vulnerability of the posterior circulation and to identify potential directions for future mechanistic and translational research.

## 2 Biological basis of endothelial senescence

### 2.1 Modes of endothelial senescence

Endothelial senescence does not arise simply as a linear consequence of chronological aging. Rather, it reflects a dynamic cellular state shaped by both replicative history and environmental stress. From a mechanistic perspective, at least two principal modes of endothelial senescence can be distinguished: replicative senescence, driven by telomere attrition and cumulative cell division, and environment-driven senescence, which is triggered prematurely by a variety of non-temporal stressors.

Replicative senescence largely reflects the replicative burden experienced by endothelial cells during long-term physiological turnover. The process generally develops gradually and is most often associated with the progressive decline in vascular function observed during normal aging (Hamczyk et al., 2020). In this sense, replicative senescence can be viewed as part of the natural aging trajectory of the vascular system (Ungvari et al., 2020).

In many pathological settings, however, endothelial senescence arises before the replicative capacity of cells is exhausted. Instead of being driven primarily by telomere shortening, senescence is frequently initiated prematurely under the influence of environmental stressors, giving rise to what is commonly referred to as environment-driven senescence.

This form of senescence typically manifests as stress-induced premature senescence (SIPS) and is closely linked to persistent or recurrent external stress signals. In endothelial cells, several environmental factors are known to promote this process, including abnormal biomechanical stimulation, disturbances in metabolic homeostasis, and chronic low-grade inflammation (Wang et al., 2022a; Ning et al., 2026; Zhang et al., 2024). Unlike senescence triggered by acute injury, stress-induced premature senescence develops gradually through the cumulative effects of sustained, low-level stress. Over time, these conditions reshape the molecular landscape and functional phenotype of endothelial cells. For this reason, environment-driven senescence is considered particularly relevant to the development of chronic vascular dysfunction.

### 2.2 Core regulatory axes of endothelial senescence

At the molecular level, the initiation and persistence of endothelial senescence are governed by a network of interacting signaling pathways. Among these, the DNA damage response (DDR) and the associated cell-cycle regulatory machinery form the central regulatory hub controlling the senescent transition of endothelial cells. Evidence from numerous *in vitro* endothelial cell studies and *in vivo* animal models indicates that sustained oxidative stress, metabolic disturbance, or repeated mechanical microinjury can lead to the gradual accumulation of DNA damage signals without triggering acute cell death. Over time, this persistent stress shifts endothelial cells from a transient adaptive state toward a stable senescent phenotype (Peng et al., 2024).

DNA damage is widely regarded as one of the most well-established initiating events in endothelial senescence. In multiple endothelial cell models, exposure to exogenous oxidative stress (such as hydrogen peroxide treatment), high-glucose conditions, or inflammatory cytokines leads to a marked increase in the DNA damage marker γH2AX, accompanied by sustained upregulation of p53 and p21 (Foret et al., 2024; Coluzzi et al., 2019; Zhou et al., 2022; Yokoi et al., 2023). These molecular changes are closely associated with G1/S cell-cycle arrest and the emergence of a senescent phenotype. Moreover, experimental suppression of p53 or p21 can partially attenuate senescence-associated features, supporting a causal role for the DDR–p53/p21 signaling axis during the initiation phase of endothelial senescence (Tai et al., 2022; Born et al., 2023).

Evidence from *in vivo* studies further supports this mechanism. In animal models of diabetes, atherosclerosis, and physiological aging, endothelial cells show persistent activation of γH2AX and p21, which correlates closely with declining vascular function (Wu et al., 2023; Xiao et al., 2021; Liu et al., 2025). These findings reinforce the central role of DNA damage signaling in the development of endothelial senescence. Notably, under conditions of chronic, low-grade stress, endothelial cells tend to favor entry into senescence rather than apoptosis, a phenomenon thought to reflect the sustained activation of low-level DDR signaling.

During the maintenance phase of senescence, the p16^INK4a/Rb pathway becomes particularly important. *In vitro* studies demonstrate that sustained upregulation of p16 through genetic manipulation can maintain endothelial cells in a senescent state even after the original stressor is removed, as indicated by persistent SA-β-gal positivity and inhibition of cell proliferation. Conversely, knockdown of p16 partially destabilizes the senescent phenotype (Grosse et al., 2020). Consistent with these findings, regions of high p16 expression in animal models frequently overlap with areas of endothelial dysfunction and vascular aging, suggesting that the p16/Rb axis plays a critical role in stabilizing and “locking in” the senescent state.

Telomere instability has also been recognized as an important molecular basis for the persistent activation of the DNA damage response. Experimental studies have shown that endothelial cells exposed to disturbed flow conditions are more susceptible to telomeric damage and exhibit a tendency toward telomere shortening (Kotla et al., 2019). Consistent with these findings, vascular regions chronically subjected to disturbed hemodynamics in human arteries display greater telomere attrition than regions exposed to laminar flow, suggesting that long-term hemodynamic conditions can influence telomere homeostasis (Chang and Harley, 1995).

It should be noted, however, that telomere shortening alone is unlikely to be sufficient to directly trigger replicative senescence. Rather, when telomere integrity is already compromised or telomere length approaches a critical threshold, abnormal flow appears to amplify senescence-associated signaling. Experimental studies have demonstrated that in mouse models with critically short telomeres, as well as in cultured endothelial cells, disturbed flow accelerates the onset of endothelial senescence through activation of the p53-p21 signaling pathway (Warboys et al., 2014). Conversely, maintenance of telomere length through exogenous enhancement of telomerase activity can attenuate the senescent phenotype induced by disturbed flow, further supporting a regulatory role for telomere dysfunction in flow-related endothelial aging (Minamino et al., 2002).

Importantly, several studies indicate that persistent telomere-associated DNA damage foci may sustain DDR signaling even when overall telomere length has not yet undergone substantial shortening. This phenomenon has been observed in both oxidative stress and mechanical stress models, suggesting that telomere dysfunction itself rather than telomere shortening *per se*, may represent a critical molecular driver of chronic endothelial senescence under conditions of sustained low-level stress (Bloom et al., 2022; Sławińska and Krupa, 2021).

DNA damage and cell-cycle arrest do not represent the end point of endothelial senescence but instead initiate a broader program of metabolic reorganization. *In vitro* studies consistently show that senescent endothelial cells exhibit impaired mitochondrial function, reduced efficiency of oxidative phosphorylation, and diminished ATP production. These changes are often accompanied by a marked reduction in the NAD^+^/NADH ratio, and declining NAD^+^ availability directly suppresses the activity of NAD^+^-dependent deacetylases such as SIRT1 (Houtkooper et al., 2012; You and Liang, 2023). In human umbilical vein endothelial cell (HUVEC) models, inhibition of SIRT1 activity exacerbates oxidative stress–induced activation of p53 and enhances the senescent phenotype (Liu et al., 2020; Wang et al., 2021). In contrast, genetic or pharmacological strategies that increase SIRT1 expression or replenish NAD^+^ precursors partially restore metabolic balance and attenuate senescence-associated changes in endothelial cells (Zhe et al., 2020; Li et al., 2022). These findings indicate that the NAD^+^–SIRT1 metabolic axis plays an active regulatory role in endothelial senescence rather than representing a secondary consequence of cellular aging.

Mitochondrial dysfunction further amplifies senescence signaling through excessive production of reactive oxygen species (ROS) (Ziegler et al., 2015; Ghosh-Choudhary et al., 2021). On one hand, ROS can directly damage both mitochondrial and nuclear DNA, thereby increasing the burden on DNA damage response pathways (Saki and Prakash, 2017). On the other hand, sustained ROS accumulation disrupts cellular redox homeostasis, reinforcing metabolic imbalance and making the senescent state more difficult to reverse (van der Reest et al., 2021). Experimental studies using antioxidant interventions or approaches that improve mitochondrial function have shown that reducing ROS levels can partially alleviate endothelial senescence, supporting a causal role for oxidative stress in amplifying senescence signaling (Zhao et al., 2025).

In addition, impairment of the autophagy–lysosome system has been implicated in the metabolic disturbances associated with endothelial aging. Both genetic and pharmacological inhibition of autophagy can induce increased ROS production and upregulation of senescence markers in endothelial cells even in the absence of overt external stress. Conversely, enhancement of autophagic flux promotes the clearance of damaged mitochondria, improves metabolic homeostasis, and delays the progression of senescence. These findings suggest that defective autophagy represents another important driver of endothelial aging (Qi et al., 2024; Tao et al., 2024).

The mechanisms described above DNA damage signaling, cell-cycle arrest, and metabolic reprogramming do not operate independently. Instead, they form a tightly interconnected regulatory network that collectively governs endothelial senescence. Experimental studies indicate that p53, in addition to serving as a central mediator of the DNA damage response, can directly influence cellular metabolism by regulating genes involved in mitochondrial biogenesis and metabolic control, such as PGC-1α. Changes in cellular metabolic status can, in turn, affect the activity and stability of p53, creating a bidirectional regulatory loop between DNA damage signaling and metabolic pathways (Vigneron and Vousden, 2010; Bloom et al., 2023a). A similar degree of interaction exists between the two principal cell-cycle regulatory pathways involved in senescence, p53/p21 and p16/Rb. Evidence from multiple senescence models suggests that impairment of one pathway often leads to compensatory activation of the other, thereby ensuring the persistence of the senescent state (Beauséjour et al., 2003). This form of redundant regulation makes endothelial senescence difficult to reverse through manipulation of a single signaling pathway once the senescence program has been established. At the functional level, the convergence of these regulatory axes ultimately manifests as a progressive loss of endothelial homeostatic control. Both *in vitro* and *in vivo* studies have shown that metabolic imbalance and accumulation of reactive oxygen species (ROS) can induce endothelial nitric oxide synthase (eNOS) uncoupling. Under these conditions, eNOS shifts from producing nitric oxide (NO) to generating additional ROS, thereby establishing a positive feedback loop between NO deficiency and oxidative stress (Han and Kim, 2023; Donato et al., 2015). This process not only impairs vasodilation and blood-flow regulation but also exacerbates DNA damage and metabolic disruption, reinforcing the persistence of senescence-associated endothelial dysfunction.

Taken together, endothelial senescence cannot be attributed to a single molecular event. Rather, it arises from the combined effects of DNA damage induced cell-cycle arrest, metabolic reprogramming, and amplification of oxidative stress mechanisms that have been repeatedly demonstrated in experimental models. Through reciprocal amplification and feedback regulation, these pathways transform transient or low-intensity environmental stress into a stable and self-sustaining senescent phenotype, which ultimately manifests at the vascular level as functional remodeling associated with endothelial aging.

### 2.3 Maintenance and amplification of endothelial senescence

Once endothelial cells enter a stable senescent state, their biological impact extends beyond intracellular changes such as cell-cycle arrest or metabolic disruption and rapidly influences the vascular wall and its surrounding microenvironment. A central mechanism underlying this transition is the SASP, which transforms endothelial senescence from a localized intracellular event into a process with broader tissue-level consequences.

Through the acquisition of SASP, senescent endothelial cells actively secrete a variety of inflammatory cytokines, chemokines, growth factors, and matrix-remodeling molecules, including IL-6, IL-8, MCP-1, and several matrix metalloproteinases (Hwang et al., 2020). Persistent secretion of these factors promotes the formation of a chronic low-grade inflammatory microenvironment within the vascular wall and amplifies senescence-associated signaling through both autocrine and paracrine mechanisms. SASP factors can directly affect neighboring endothelial cells, inducing functional impairment and even secondary senescence (Giu et al., 2023; Fan et al., 2024). At the same time, they influence the behavior of vascular smooth muscle cells and immune cells, promoting inflammatory responses and structural remodeling within the vessel wall, thereby extending the spatial impact of endothelial aging (Pantsulaia et al., 2016; Ruscetti et al., 2020). An important implication of SASP activity is that endothelial senescence acquires a propagative character. The senescent phenotype is no longer restricted to the originally affected cells but can gradually spread through microenvironmental signaling. This paracrine amplification mechanism is thought to contribute to the progressive increase in the proportion of senescent cells observed in chronic vascular dysfunction. Moreover, the inflammatory microenvironment generated by SASP can further enhance oxidative stress and increase the burden of DNA damage, thereby reinforcing activation of DDR signaling and other senescence-related pathways and establishing a self-sustaining positive feedback loop.

Beyond SASP-mediated signaling amplification, epigenetic dysregulation provides a deeper molecular basis for the long-term stability of endothelial senescence. Senescent endothelial cells commonly exhibit widespread alterations in DNA methylation patterns, specific histone modifications, and remodeling of chromatin architecture. These changes effectively lock in senescence-associated transcriptional programs, allowing cells to maintain the senescent phenotype even after the original external stress has diminished or been removed (Horvath, 2013; Chao et al., 2024). Such epigenetic “memory” not only stabilizes the senescent state but also alters how endothelial cells respond to subsequent stimuli. Compared with young or non-senescent endothelial cells, senescent cells display heightened sensitivity to mechanical stress, metabolic fluctuations, and inflammatory signals. As a result, stress- and inflammation-related signaling pathways are more readily activated, further accelerating endothelial dysfunction.

At the tissue level, the combined effects of the SASP and epigenetic dysregulation gradually shift the vascular microenvironment away from homeostasis, creating conditions that favor the persistence and spread of cellular senescence. Altered activity of epigenetic enzymes appears to play an important regulatory role in this process. For example, changes in the expression of histone methyltransferases (HMTs) have been shown to markedly suppress the proliferative and migratory capacity of vascular smooth muscle cells (VSMCs), thereby influencing the dynamic remodeling of the vascular wall. In endothelial cells, inflammatory stimulation can trigger extensive epigenetic remodeling. Genome-wide methylation analyses have shown that exposure of endothelial cells to TNF-α induces significant methylation changes in multiple genes involved in cell adhesion, barrier permeability, the renin–angiotensin system, and eNOS regulation. These findings suggest that inflammatory signaling can reshape endothelial functional phenotypes through epigenetic mechanisms (Milenkovic et al., 2020).

ROS further intensify this process by modulating the activity of DNA methyltransferases. Experimental studies have demonstrated that ROS can directly upregulate DNMT1, promoting aberrant methylation of inflammation-related genes and establishing a reinforcing feedback loop between senescence and inflammation in endothelial cells. In addition, excessive methylation mediated by DNMT1 and DNMT3A can affect transcription factors involved in mechanosensing and flow-responsive signaling, including homeobox protein A5 (HOXA5) and Krüppel-like factors 3 and 4 (KLF3, KLF4). Through these mechanisms, epigenetic dysregulation may weaken the ability of endothelial cells to adapt to physiological shear stress and amplify functional disturbances caused by disturbed flow (Dunn et al., 2015).

Against this backdrop, the sustained presence of inflammatory mediators and matrix-remodeling molecules released through SASP further alters the composition and mechanical properties of the extracellular matrix. These changes modify the patterns of shear and tensile stress experienced by endothelial cells. Persistent alterations in the microenvironment can continuously stimulate senescent endothelial cells while simultaneously promoting stress responses and senescence in neighboring, previously unaffected endothelial cells through both paracrine signaling and mechanical cues. Over time, this process contributes to the accumulation of senescent cells within the vascular wall and establishes a self-reinforcing cycle of progressive endothelial dysfunction.

### 2.4 From endothelial cell senescence to vascular endothelial aging

As senescent endothelial cells gradually accumulate within the vascular wall, their impact extends beyond dysfunction at the level of individual cells and begins to reshape the overall functional state of the endothelium. This transition gives rise to vascular endothelial aging. Unlike conventional concepts of endothelial dysfunction, vascular endothelial aging is not defined by persistent abnormalities in a single functional parameter. Rather, its hallmark lies in a progressive contraction of the dynamic regulatory range through which blood vessels respond to physiological and pathological stimuli. As a result, vascular regulation shifts from a flexible and adaptive process toward responses that are slower, less efficient, and less capable of adjusting to changing conditions.

One of the most characteristic changes during this process is the decline in nitric oxide (NO) synthesis and bioavailability. As the proportion of senescent endothelial cells increases, both the expression and activity of endothelial nitric oxide synthase (eNOS) become suppressed through multiple mechanisms. At the same time, elevated oxidative stress accelerates NO degradation. Consequently, when vessels are exposed to changes in shear stress or increased metabolic demand, they are often unable to generate an adequate or timely vasodilatory response (Jin et al., 2019; Harvey et al., 2015). Importantly, the resulting impairment in NO-dependent regulation does not necessarily manifest as sustained vasoconstriction; rather, it is more commonly reflected in a reduced capacity of blood vessels to respond effectively to rapid or complex hemodynamic fluctuations (Zhang et al., 2025a5; Ishihata et al., 2006).

At the level of vascular tone regulation, endothelial aging is typically characterized by an imbalance between diminished vasodilatory signaling and relatively enhanced vasoconstrictive influences. Although vessels may maintain an apparently normal diameter and baseline blood flow under resting conditions, their ability to adapt to physiological challenges such as postural changes, fluctuations in flow direction, or transient variations in perfusion pressure becomes markedly reduced (Kong et al., 2016). In addition, inflammatory mediators released through SASP can interfere with the finely tuned signaling between endothelial cells and vascular smooth muscle cells, further increasing the heterogeneity and instability of vascular responses (Yang et al., 2024).

Beyond abnormalities in vasomotor control, endothelial aging also compromises vascular barrier integrity by disrupting intercellular junctions. Altered expression of tight junction and adherens junction proteins in senescent endothelial cells weakens the structural integrity of the vascular barrier, allowing inflammatory mediators and metabolic by-products to cross the vessel wall more readily into surrounding tissues (Propson et al., 2021; Zhan et al., 2023; Jiang et al., 2024). Increased vascular permeability not only intensifies local inflammation and oxidative stress but also feeds back to further impair endothelial function, creating a self-reinforcing cycle that promotes the progression of endothelial aging.

**FIGURE 1:**
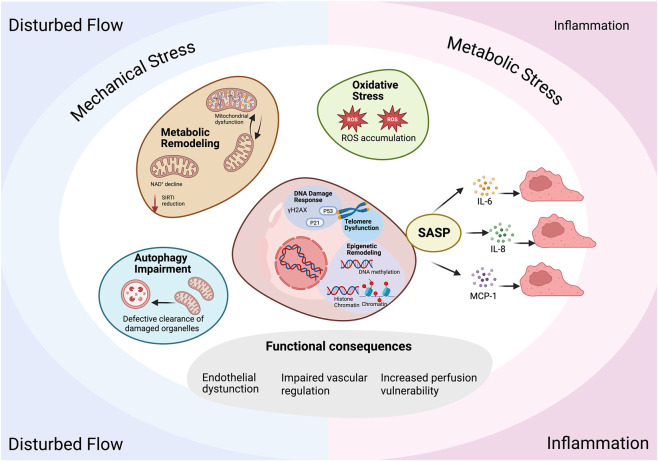
Mechanistic network underlying endothelial senescence and its functional consequences. Endothelial senescence arises from both replicative exhaustion and environment-driven stress, including disturbed flow, mechanical stress, metabolic imbalance, and chronic inflammation. These stimuli converge on the activation of the DNA damage response characterized by γH2AX accumulation and p53/p21 signaling, and are further reinforced by telomere dysfunction. Metabolic remodeling, including mitochondrial dysfunction, NAD^+^ decline, and reduced SIRT1 activity, promotes the accumulation of ROS, which in turn amplifies DNA damage and senescence signaling. Impairment of autophagy further contributes to the persistence of damaged organelles and oxidative stress.

These multilayered functional alterations become particularly evident in vascular beds characterized by complex anatomical structures and highly variable hemodynamic conditions. In such regions, the reduced regulatory capacity, delayed responsiveness, and impaired barrier stability associated with endothelial aging may interact and amplify one another. The net effect is a heightened sensitivity of tissue perfusion to external disturbances what may be described as increased perfusion vulnerability. Under these conditions, even in the absence of overt vascular stenosis or acute occlusion, relatively minor changes in blood flow, body posture, or mechanical environment may be sufficient to trigger measurable perfusion deficits and functional impairment (Figure 1).

Once established, senescent endothelial cells acquire a senescence-associated secretory phenotype (SASP), leading to sustained inflammatory signaling and paracrine propagation of senescence. In parallel, epigenetic remodeling stabilizes senescence-associated transcriptional programs, reinforcing the persistence of the senescent state.

Collectively, these interconnected processes result in endothelial dysfunction, characterized by reduced nitric oxide (NO) bioavailability, impaired vasoregulatory capacity, and increased vulnerability of vascular perfusion to external stress.

## 3 Disturbed flow and endothelial senescence

The vascular endothelium is not a passive barrier that merely withstands blood flow. Rather, it is a highly specialized mechanosensory interface that converts shear stress, tensile strain, and pressure fluctuations into specific intracellular signals. Under physiological conditions, this mechano-biological signaling helps preserve endothelial homeostasis. Under conditions of abnormal or chronically disturbed flow, however, the same sensing system may continuously activate pro-senescent pathways and thereby become an important external driver of endothelial aging. It should be emphasized that endothelial responses to mechanical stimulation are determined not only by whether flow is disturbed, but also by the magnitude of shear stress itself. Previous studies have shown that stable laminar shear stress within the physiological range generally maintains anti-inflammatory, antioxidant, and antithrombotic phenotypes, whereas low shear stress is more often associated with inflammatory activation, enhanced oxidative stress, and accumulation of senescence-related features. By contrast, excessively high shear stress may trigger adaptive responses in the short term but can also lead to endothelial injury or maladaptive remodeling when sustained. Thus, the mechanosensory and mechanotransductive network itself constitutes a major regulatory interface in endothelial senescence (Cheng C. K. et al., 2025; Wu et al., 2025).

Under physiological laminar shear stress, endothelial cells engage a highly conserved shear-dependent transcriptional program that translates mechanical stimuli into homeostatic molecular signals. Myocyte enhancer factor 2 (MEF2) is a shear-sensitive transcription factor that serves as a key upstream hub linking mechanical input to transcriptional regulation. Under laminar flow, particularly stable unidirectional shear stress within the physiological range (approximately 10–20 dyn/cm^2^), MEF2 activity increases and directly induces the transcription of Krüppel-like factor 2 (KLF2) and KLF4, thereby initiating an endothelial homeostatic program (Sacilotto et al., 2016; Lu et al., 2021; Kumar et al., 2005). KLF2 and KLF4 are widely recognized as central homeostatic transcription factors in endothelial cells. Their expression strongly influences whether the endothelium maintains an anti-inflammatory and antioxidant phenotype or shifts toward stress-associated and pro-senescent states (Salmon et al., 2025; Li Z. Y. et al., 2023). Functional studies have shown that KLF2/KLF4 upregulate multiple anti-inflammatory, antioxidant, and antithrombotic genes while suppressing persistent activation of inflammatory and stress-related pathways (Joshi et al., 2024; Cho et al., 2025; Li S. et al., 2024). Through this mechanism, KLF2/KLF4 not only reduce excessive reactive oxygen species generation under metabolic fluctuation or oxidative stress, but also restrain activation of DNA damage responses and cell-cycle arrest signals, thereby increasing endothelial tolerance to chronic stress (T et al., 2019; Zhang et al., 2021). Flow-based experimental studies have further demonstrated that MEF2 is required for laminar-flow-induced KLF2 and KLF4 expression, and that sustained activation of the MEF2–KLF2/KLF4 axis is essential for interpreting mechanical signals as homeostatic rather than pro-senescent inputs (Lu et al., 2021; Lee et al., 2024; Tamargo et al., 2024).

By contrast, under low shear stress (typically <4 dyn/cm^2^), KLF2/KLF4 expression can be suppressed even in the absence of overt acute injury, with a concomitant decline in endothelial anti-inflammatory and antioxidant capacity. This protective pathway is further weakened when low shear coexists with oscillatory flow. In studies of aortic bifurcations and curved vascular segments, local low-shear environments have consistently been associated with sustained inflammatory signaling, increased oxidative stress, and upregulation of senescence-related markers. These observations indicate that the MEF2–KLF2/KLF4 axis is sensitive not only to flow stability but also to whether shear stress remains within a protective range. Its integrity therefore plays a central role in determining whether endothelial cells can maintain a non-senescent state under long-term mechanical stimulation.

Under oscillatory or disturbed flow, this homeostatic checkpoint is progressively disrupted. Class IIa histone deacetylases, particularly HDAC5 and HDAC7, are highly sensitive regulators of flow pattern, and their subcellular localization directly influences the integrity of the endothelial homeostatic transcriptional program (Travers et al., 2020; Brancolini, 2025). A large body of experimental work has shown that disturbed flow induces nuclear translocation of HDAC5/7, where they form inhibitory complexes with MEF2 and suppress MEF2-dependent transcription of KLF2 and KLF4 (Ginnan et al., 2012; Liu et al., 2009; Wang et al., 2010; Cho et al., 2026). Importantly, HDAC5/7 do not directly activate pro-inflammatory or pro-senescent genes. Rather, they shut down the transcriptional program that normally maintains endothelial anti-inflammatory, antioxidant, and stress-resistant functions, thereby releasing multiple stress pathways from long-term suppression. Low shear stress is not a negligible background factor in this setting. Instead, it often coexists with oscillatory shear to create a more pathogenic mechanical environment. Under these conditions, the already reduced KLF2/KLF4 background is further weakened, allowing low-intensity stress signals that would normally be buffered to persist. This HDAC5/7-mediated transcriptional reprogramming does not immediately cause cell death or overt structural injury. Instead, it gradually impairs the endothelial capacity to regulate chronic low-grade stress and favors a shift toward cell-cycle arrest and functionally conservative adaptation. This pattern is consistent with the molecular features of endothelial senescence and may therefore represent a critical step by which mechanical signaling shifts from homeostatic input to pro-senescent input. In the vertebral artery, where flow can fluctuate over time and across segments with posture and motion, HDAC5/7-mediated transcriptional repression may occur as repeated episodes of activation and partial recovery. If accompanied by local flow-velocity reduction and low shear stress, this effect may accumulate over time and progressively diminish endothelial resilience to chronic stress.

Once this homeostatic transcriptional barrier is weakened, the mechanosensitive transcriptional regulators Yes-associated protein (YAP) and its paralog TAZ become major amplifiers of long-term endothelial phenotypic remodeling. Multiple *in vitro* studies using oscillatory shear models have shown sustained nuclear localization of YAP/TAZ under disturbed flow. Their activation depends on enhanced integrin–FAK–SRC signaling and suppression of the Hippo pathway kinase cascade (Park et al., 2021; Shyy and Chien, 2002). This mechanically driven regulation places YAP/TAZ at the interface between abnormal flow patterns and transcriptional reprogramming. Unlike the reversible transcriptional changes induced by brief mechanical stimulation, sustained activation of YAP/TAZ can profoundly alter how endothelial cells respond to environmental cues (Wang et al., 2016). Functional studies further demonstrate that genetic knockdown or pharmacological inhibition of YAP/TAZ attenuates inflammatory responses and senescence-associated markers, including increased p21 expression and SA-β-gal activity, under disturbed flow (Cao et al., 2023; Sladitschek-Martens et al., 2022; Barettino et al., 2024). These findings indicate that YAP/TAZ are not passive bystanders but necessary amplifiers and maintainers of flow-induced senescence. Moreover, YAP/TAZ do not act solely on inflammatory genes. They directly participate in transcriptional regulation of core senescence networks. Previous studies have shown functional coupling between YAP/TAZ and the p53/p21 axis, as well as transcriptional control of multiple senescence-associated secretory phenotype (SASP) genes (Zinatizadeh et al., 2021; Xu et al., 2025). This pathway is sensitive not only to flow pattern but also to cytoskeletal tension, matrix stiffness, and external mechanical compression. Its activation therefore cannot be reduced to disturbed flow alone. Under low shear conditions, cytoskeletal remodeling and mechanical instability may further enhance YAP/TAZ signaling. Under sustained high mechanical loading or persistent external compression, a different form of maladaptive remodeling may emerge. In the vertebral artery, which is exposed to vascular curvature, tensile strain, and local compression, YAP/TAZ are therefore more likely to integrate multiple mechanical inputs. This may produce regulatory features distinct from those observed in single-shear-force models and may drive endothelial cells from reversible mechanical adaptation toward a more stable senescent phenotype.

At the same time, disturbed flow amplifies oxidative stress through metabolic regulators, thereby accelerating pro-senescent signaling. Under oscillatory shear stress, endothelial NOX2 and NOX4 expression is markedly increased, while eNOS becomes uncoupled, shifting reactive oxygen species generation from signaling-level production to sustained accumulation (Zheng et al., 2022). This process is closely linked to suppression of metabolic sensing pathways such as AMPK and SIRT1 (Cronin et al., 2024). Several studies have shown that activation of AMPK or enhancement of SIRT1 activity can partially reverse oscillatory-shear-induced oxidative stress and endothelial senescence, indicating that the metabolic axis acts as an amplifier in the coupling between mechanics and senescence (Liang et al., 2025). Low shear stress itself may also serve as a permissive background for oxidative amplification. On the one hand, low shear reduces expression of endothelial protective genes and weakens the capacity to buffer ROS. On the other hand, when low shear coexists with oscillatory flow, NOX-dependent oxidative signaling and eNOS uncoupling are more likely to persist. From the perspective of metabolism and oxidative stress, the amplification of senescence-related stress is therefore not restricted to disturbed flow alone, but may be even more pronounced in a combined environment of low shear and disturbed flow.

With sustained oxidative stress and metabolic imbalance, endothelial cells gradually enter a state characterized by chronic low-level activation of the DNA damage response. Unlike acute injury, disturbed flow is more commonly associated with persistent but moderate activation of the ATM/ATR–p53–p21 axis, a signaling pattern that favors senescence rather than apoptosis. Further studies have shown that upregulation of DNMT1 and DNMT3A, together with accumulation of repressive histone marks such as H3K9me3 and H3K27me3, can stabilize a senescence-associated transcriptional profile at the epigenetic level, keeping mechanosensing and homeostatic genes in a suppressed state (Pinto et al., 2024; Jiang et al., 2015). Even when laminar shear is subsequently restored, endothelial cells may remain highly sensitive to inflammatory and stress-related signals and continue to display a senescent phenotype (Lu et al., 2019). These findings suggest that chronic mechanical abnormalities do not act only as transient insults, but can be converted into long-lasting cellular states through DNA damage signaling and epigenetic memory. In the vertebral artery, where low shear stress and directional flow fluctuation may recur because of vascular curvature, postural changes, or dynamic compression, this repetitive-stress-to-stable-phenotype transition may be particularly relevant.

It should also be noted that the major evidence supporting the mechanotransduction pathways discussed above comes from studies of endothelial cells derived from the aorta, carotid artery, and coronary artery. These vascular beds typically have relatively straight courses and more stable flow patterns, and their endothelial cells are exposed predominantly to shear-dominated mechanical stimulation. By contrast, the vertebral artery differs substantially in both anatomical configuration and biomechanical exposure. Its segments course through the transverse foramina of the cervical spine, follow a tortuous path, and are continuously subjected to tensile strain, torsion, and local compression during cervical flexion, extension, and rotation (Mitchell, 2003; Li et al., 1999; Hong et al., 2022). Accordingly, the mechanical signals sensed by vertebral artery endothelial cells are unlikely to arise from shear stress alone, but rather from a combination of multiple inputs.

Within this context, the functional states of these mechanotransduction pathways may undergo context-dependent modification. First, from the perspective of mechanical sensitivity, chronic external constraint may alter the response threshold of endothelial cells to shear stress, thereby shifting their biological response to the same hemodynamic stimulus (Hahn and Schwartz, 2009). For example, a shear signal that preserves homeostasis in conventional models, such as KLF2-mediated anti-inflammatory signaling, may fail to maintain the same protective effect in a composite mechanical environment (Dekker et al., 2002). Second, from the perspective of spatial force distribution, local confinement by the transverse foramina may generate heterogeneous tension fields across different regions of the vessel wall. Such mechanical discontinuity may affect cytoskeletal tension and nuclear structural stability and may in turn contribute to abnormal activation of force-sensitive pathways such as YAP/TAZ (Dupont et al., 2011). In light of these differences, the pathways described above should be regarded not as directly transferable mechanisms for the vertebral artery, but as a broadly relevant mechanobiological framework whose specific functional states may be substantially modulated by vascular geometry, mechanical constraint, and dynamic motion. In the present review, these mechanisms are therefore used to construct a plausible link between hemodynamic disturbance and endothelial senescence, rather than to claim a pathway that has already been directly validated in the vertebral artery.

**FIGURE 2:**
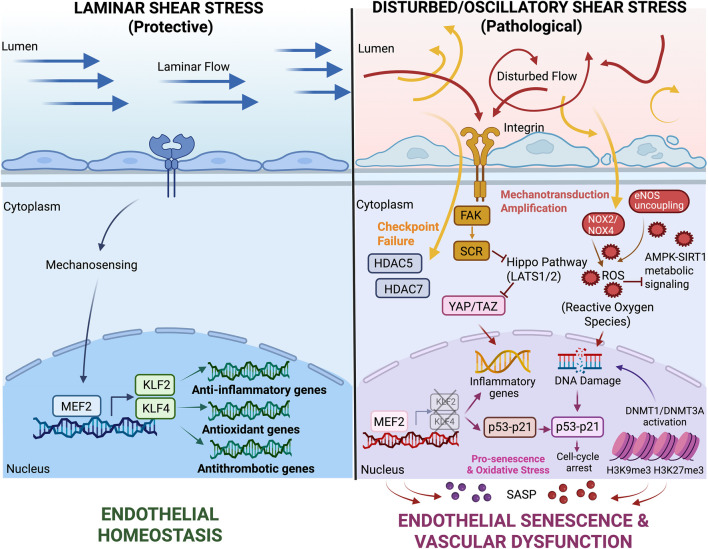
Disturbed shear stress drives endothelial senescence:Laminar shear stress activates the MEF2–KLF2/KLF4 pathway to maintain endothelial homeostasis. In contrast, disturbed flow suppresses this protective signaling through HDAC5/7 and activates integrin–FAK–SRC–YAP/TAZ pathways, promoting oxidative stress, DNA damage, and p53–p21–mediated cell-cycle arrest, ultimately leading to endothelial senescence and vascular dysfunction.

In summary, biomechanical stress and disturbed flow drive endothelial senescence through a multistage process rather than through any single signaling pathway. This process involves altered interpretation of mechanical signals, downward resetting of homeostatic thresholds, transcriptional and metabolic amplification, and eventual epigenetic stabilization. Importantly, it is governed not only by flow pattern but also by shear stress magnitude. Stable laminar shear within the physiological range supports endothelial homeostasis, low shear more readily cooperates with oscillatory flow to promote chronic stress and accumulation of senescence-related phenotypes, and persistently excessive shear may induce a different form of injury or remodeling response. Nevertheless, although these mechanisms have been supported by substantial experimental evidence in the aorta, carotid artery, and other vascular systems, direct evidence that cervical degeneration drives endothelial senescence in the vertebral artery through these pathways is still lacking. The mechanistic link proposed here should therefore be interpreted as a hypothesis-driven framework derived from established vascular biology and the specific anatomical and mechanical features of the vertebral artery. Its applicability to the vertebral artery remains to be tested in future experimental and clinical studies (Figure 2).

## 4 Vertebral artery microenvironment remodeling in cervical degeneration

The vertebral artery is a medium-caliber vessel with a complex course and close structural coupling to the surrounding osseous anatomy. Even under physiological conditions, its hemodynamic environment shows marked spatial and temporal heterogeneity. Unlike vessels embedded in relatively stable soft-tissue surroundings, the vertebral artery ascends through multiple cervical transverse foramina. Its geometry, curvature, and mechanical loading are therefore continuously influenced by cervical anatomy and motion. As a result, the vertebral artery exists within a flow-regulatory environment that is inherently dependent on both anatomical configuration and biomechanical context.

As cervical degeneration progresses, several changes occur. The disc height decreases. The bones remodel. The movement of each segment changes. These changes gradually alter the mechanical properties of the vascular and osseous system. Importantly, these changes should not be equated with acute vascular injury or overt structural stenosis. Rather, they are more likely to manifest as chronic remodeling of vascular geometry, local mechanical constraint, and hemodynamic state. In this setting, the vertebral artery is more likely to experience low-amplitude, persistent, and recurrent flow perturbations than isolated severe events. It is under these anatomical and mechanical conditions that the vertebral artery provides a distinctive setting in which to examine how hemodynamic abnormalities may reshape the vascular microenvironment over time.

Unlike large arteries situated in relatively stable soft-tissue environments, the vertebral artery is tightly coupled to the osseous structures of the cervical spine and is continuously influenced by both static anatomical constraints and motion-related forces. In this context, cervical degeneration should not be viewed simply as a direct cause of endothelial senescence. Rather, it is better understood as a chronic structural and biomechanical condition that may reshape local hemodynamics and the cellular microenvironment, thereby creating a permissive background for endothelial dysfunction and the development of senescence-related phenotypes.

### 4.1 Anatomical and biomechanical alterations

The vertebral artery exhibits a highly segmental and spatially dependent anatomy along the cervical spine. Its cervical course is typically divided into several anatomical segments, including the origin segment, the transverse foraminal segment, and the atlantoaxial segment. Among these, the portion passing through the transverse foramina of the cervical vertebrae is most tightly coupled to surrounding osseous structures (Magklara et al., 2021; Madonis and Jenkins, 2021) Unlike arteries embedded primarily within soft tissues, this segment is constrained by adjacent vertebral bodies, facet joints, and ligamentous structures even under normal anatomical conditions, resulting in inherent limitations in both its geometric configuration and spatial mobility (Mitchell; Gailloud, 2022).

During the progression of cervical degeneration, gradual loss of intervertebral disc height and structural alterations of the annulus fibrosus may lead to subtle but persistent adjustments in the spatial relationships between adjacent vertebrae. These changes affect not only vertebral alignment but may also modify the three-dimensional orientation and relative positioning of the transverse foramina. As a result, the pathway through which the vertebral artery traverses the cervical spine may be indirectly altered (Gul and Atik, 2024; Zibis et al., 2018).At the same time, osseous remodeling along vertebral margins and facet joint regions can modify the spatial boundaries surrounding the artery. In certain segments, this may lead to increased vascular curvature or displacement from its typical course (Byun et al., 2022). Importantly, the vertebral artery does not exist in isolation within the transverse foramen. It is accompanied by a venous plexus, sympathetic nerve fibers, and surrounding connective tissue. This complex anatomical environment implies that even in the absence of direct luminal compression, subtle alterations in surrounding tissues may influence the mechanical support and buffering capacity of the vessel, thereby modifying how the artery experiences mechanical forces during cervical motion.

Beyond static anatomical factors, changes in segmental motion patterns of the cervical spine represent another key determinant of vertebral artery biomechanics. As degenerative processes progress, the distribution of motion across cervical segments may be redistributed: mobility may decrease in certain segments while adjacent segments undergo compensatory increases in motion (Xia et al., 2021). During cervical flexion, extension, rotation, and combined movements, such changes can lead to uneven distribution of tensile, torsional, and bending stresses along different portions of the vertebral artery (Sato et al., 2023; Zhang et al., 2016).

The cumulative effects of these structural and kinematic alterations are reflected in the progressive remodeling of vertebral artery geometry. This may include increased vascular curvature, changes in local angulation, and reduced geometric continuity along the vessel course. Importantly, these changes rarely manifest as a single discrete site of stenosis or obstruction. Instead, they tend to create distributed spatial constraints along extended segments of the artery, allowing flow patterns to fluctuate dynamically over time.Within this anatomical and biomechanical context, the vertebral artery becomes increasingly exposed to a complex and fluctuating mechanical environment, providing a structural basis for the gradual remodeling of the vascular microenvironment over chronic time scales.

### 4.2 Hemodynamic alterations

Within the anatomical and biomechanical context described above, cervical degeneration may influence not only flow patterns in the vertebral artery but also the magnitude of shear stress, both of which are key determinants of endothelial phenotype. Unlike large arteries with relatively stable courses, vertebral artery flow is strongly shaped by vessel geometry, segmental osseous constraints, and cervical motion.

A number of dynamic studies indicate that vertebral artery flow is not constant but exhibits clear position and segment-dependent variability. During neck rotation, extension, or combined movements, transient reductions in flow velocity have been reported. In certain pathological conditions, transient flow interruption, reversal, or reversible occlusion due to dynamic compression may also occur (Arnold et al., 2004).

In addition to motion-related changes, cervical degeneration itself may be associated with reduced vertebral artery hemodynamics. Previous Doppler studies have shown that the severity of degeneration, abnormal cervical curvature, and related structural changes may correlate with decreased flow parameters. These findings suggest that, under degenerative conditions, the vertebral artery is more likely to experience chronic, low-amplitude, and recurrent hemodynamic disturbances. It should be noted, however, that most of these studies report flow velocity, volume, or related indices rather than direct measurements of local wall shear stress. Thus, the inference of “low shear” is primarily based on reduced flow velocity, dynamic compression, and complex vessel geometry, rather than on direct *in situ* measurements (Shende et al., 2023; Bayrak et al., 2009).

From a hemodynamic perspective, reduced flow velocity, position-dependent fluctuations, and local dynamic compression together suggest that vertebral artery endothelial cells may be repeatedly exposed to a composite environment characterized by unstable flow direction and reduced effective shear stress. Based on mechanisms established in other vascular beds, such conditions are generally associated with attenuation of protective endothelial signaling and enhancement of stress-related pathways, particularly suppression of KLF2/KLF4-mediated programs and sustained activation of oxidative stress. However, the precise activation of these pathways in the vertebral artery has not been directly demonstrated. In this review, these mechanisms are therefore presented as a conceptual interpretation of observed hemodynamic features, rather than as vertebral artery specific conclusions (Arnold et al., 2004).

Importantly, this hemodynamic state differs from that of acute ischemic events. The vertebral artery is more likely to experience chronic, low-grade, and recurrent fluctuations in flow and shear stress, rather than a single episode of severe perfusion interruption. Over time, such sub-threshold but persistent mechanical stress may provide a more plausible basis for the gradual development of endothelial dysfunction and senescence-related phenotypes.

### 4.3 Chronic hypoperfusion and metabolic stress

Within the anatomical and biomechanical context associated with cervical degeneration, blood flow through the vertebral artery is more likely to exhibit long-term, low-amplitude reductions in perfusion accompanied by fluctuating flow patterns. This hemodynamic state differs fundamentally from the acute ischemic injury caused by vascular occlusion or severe transient stenosis. Instead, it resembles a persistent hypoperfusion environment that remains below the threshold for overt tissue necrosis (Ohnishi et al., 2021; Macnab, 1975). Experimental studies suggest that such conditions often do not induce immediate cell death but can gradually reshape the metabolic and functional state of vascular wall cells over time.

Chronic hypoperfusion first imposes a sustained challenge to endothelial energy homeostasis. In contrast to the rapid energy depletion observed during acute ischemia, prolonged reductions in perfusion tend to trigger metabolic adaptation, allowing endothelial cells to maintain basic homeostatic functions under conditions of limited energy supply (Han et al., 2020). In multiple models of hypoperfusion and hypoxia, endothelial mitochondria show a progressive decline in oxidative phosphorylation efficiency and metabolic flexibility. These findings suggest that mitochondrial function is not completely lost but instead shifts toward a lower-efficiency, energy-conserving metabolic state (Su et al., 2022; He et al., 2024).

Beyond changes in mitochondrial respiration, chronic hypoperfusion may also influence mitochondrial dynamics (Du et al., 2024; Feng et al., 2018). The balance between mitochondrial fusion and fission is essential for maintaining metabolic efficiency and cellular stress tolerance (Kawano et al., 2023; Chen et al., 2025; Toyama et al., 2016). Under conditions of prolonged energy limitation and fluctuating perfusion, endothelial mitochondrial networks tend to become increasingly fragmented. Although this structural pattern may facilitate short-term survival by isolating damaged components, it often comes at the cost of reduced metabolic efficiency and functional flexibility, thereby increasing the overall burden of metabolic stress (Zhang et al., 2025b).

At the same time, the global metabolic phenotype of endothelial cells may shift. Under physiological conditions, endothelial cells already rely heavily on glycolysis for energy production. During chronic hypoperfusion, this metabolic preference may become further reinforced, gradually reducing the ability of cells to switch flexibly between metabolic pathways in response to changing energy availability (Jiang et al., 2023; Alhusban et al., 2024). Such metabolic reprogramming does not necessarily lead to immediate functional failure. However, it substantially narrows the range and speed of endothelial adaptive responses to environmental fluctuations.

As a central node linking energy metabolism, redox balance, and stress regulation, NAD^+^ homeostasis is also vulnerable to disruption in the setting of chronic hypoperfusion. Persistent reductions in perfusion and ongoing metabolic stress can disturb the balance between NAD^+^ synthesis and consumption, resulting in a gradual decline in intracellular NAD^+^ levels (Zhao et al., 2021; Wang et al., 2024). The consequence is not simply diminished energy production but rather a reduction in the metabolic reserve capacity required for endothelial cells to cope with additional oxidative or metabolic challenges.At the level of cellular quality control, autophagy is similarly affected by chronic hypoperfusion. Under conditions of energy limitation and cellular stress, autophagy plays a crucial role in removing damaged mitochondria and abnormal protein structures. However, when hypoperfusion persists over extended periods, autophagy may shift from an early compensatory activation toward a state of impaired flux, leading to reduced efficiency in the clearance of damaged cellular components (Wang et al., 2023; Kang et al., 2023). In this context, autophagic function is not entirely lost but becomes mismatched with the level of cellular stress, thereby further increasing the burden on endothelial homeostatic regulation (Tian et al., 2024; Takagaki et al., 2020).

Importantly, metabolic stress under chronic hypoperfusion also exhibits a distinct temporal dimension. Unlike persistent severe hypoxia, this environment more often reflects an imbalance between oxygen supply and metabolic demand, with perfusion levels fluctuating periodically according to body posture and activity state (Badran et al., 2014; Butcher et al., 2013). This temporal heterogeneity places endothelial cells in a prolonged state of stress readiness rather than allowing full adaptation or recovery, thereby increasing the likelihood of regulatory failure.

Taken together, chronic hypoperfusion and metabolic stress form a critical metabolic foundation for the remodeling of the vertebral artery microenvironment. Through long-term alterations in mitochondrial function, metabolic phenotype, NAD^+^ homeostasis, and autophagic regulation, endothelial cells are maintained in a metabolically strained yet still viable state. Without causing overt acute injury, this condition provides a permissive background for the persistent presence of local inflammatory signaling and the gradual accumulation of senescence-associated phenotypes within the vascular wall.

### 4.4 Inflammaging in the vertebral artery microenvironment

Within the anatomical and perfusion context associated with cervical degeneration, inflammatory activity within the vertebral artery wall often displays pronounced localization and temporal persistence. Unlike systemic inflammatory responses or classical forms of vasculitis, this state is not typically triggered by a single acute stimulus. Rather, it resembles a form of chronic inflammatory microenvironment with aging-related features, in which the defining characteristic is not the intensity of inflammation but the prolonged presence and incomplete resolution of inflammatory signaling within the vascular wall (Higashi and Taguchi, 2025; Pacinella et al., 2022).

The vertebral artery in the cervical region travels through relatively confined spaces formed by osseous and connective tissue structures. Within this anatomical setting, the diffusion, clearance, and dilution of inflammatory mediators are spatially constrained (Froom et al., 2025). As a result, even modest levels of inflammatory mediator production may exert biological effects over extended periods within the local microenvironment (Lawrence and Gilroy, 2007). This spatially restricted inflammatory context provides a structural basis for the gradual accumulation and persistence of inflammatory signals over time, predisposing the vascular microenvironment to a low-grade, senescence-like inflammatory state.

Within such a local inflammatory environment, endothelial cells tend to adopt a functional phenotype characterized by sustained low-level expression of inflammatory mediators. In this setting, the senescence-associated secretory phenotype (SASP) does not manifest as a large-scale acute release of inflammatory cytokines. Instead, it operates primarily through persistent, low-intensity paracrine signaling. The continued presence of these SASP-related signals gradually shifts the vascular inflammatory state from a reversible stress response toward a more stable form of inflammatory aging (Tellides and Pober, 2015; Li H. et al., 2023).

Importantly, the persistence of this inflammatory microenvironment does not depend on abnormal activation of a single cell type. Rather, it emerges from the collective participation of multiple vascular wall cells under chronic stress conditions. Endothelial cells, vascular smooth muscle cells, and perivascular supporting cells interact within this environment to form a network of reciprocal signaling. Through these interactions, inflammatory signals can be locally amplified and sustained, leading to a vascular microenvironment characterized by diminished regulatory capacity and reduced resilience to physiological perturbations (Li X. et al., 2024; Mahoney et al., 2024; Cheng Y. et al., 2025; Zhang et al., 2025c).

In addition, cervical degeneration typically occurs during life stages in which tissue plasticity and repair capacity gradually decline. This systemic aging context further favors the transition of local inflammatory activity toward senescence-associated phenotypes (Badhiwala et al., 2020). Under such conditions, even when external stimuli do not continue to increase, inflammatory signaling within the vertebral artery wall may persist for extended periods, gradually stabilizing into a chronic, low-grade inflammatory state that is difficult to fully reverse.

**FIGURE 3:**
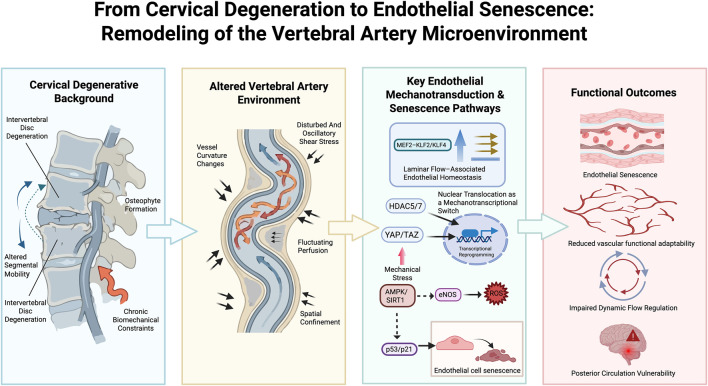
From cervical degeneration to endothelial senescence: remodeling of the vertebral artery microenvironment. This schematic illustrates how chronic structural and biomechanical changes associated with cervical spine degeneration reshape the vertebral artery microenvironment and ultimately promote endothelial senescence and functional vulnerability of the posterior circulation. Cervical degenerative changes, including intervertebral disc degeneration, osteophyte formation, and altered segmental mobility, impose persistent biomechanical constraints on the vertebral artery. These structural conditions lead to alterations in vascular geometry, spatial confinement, and repetitive fluctuations in shear stress and perfusion. Under these conditions, endothelial mechanotransduction pathways—including the MEF2–KLF2/KLF4 axis, HDAC5/7 nuclear translocation, YAP/TAZ activation, and metabolic stress signaling involving AMPK/SIRT1, eNOS, and reactive oxygen species—contribute to transcriptional reprogramming and the stabilization of endothelial senescence through p53/p21 signaling. The cumulative effect of these processes is a progressive decline in vascular functional adaptability, impaired dynamic flow regulation, and increased vulnerability of the posterior circulation.

In summary, cervical degeneration may give rise to a local vascular environment characterized by disturbed flow patterns, reduced shear stress, chronic metabolic stress, and persistent low-grade inflammation. Based on well-established mechanisms in other vascular systems, such conditions are considered permissive for endothelial dysfunction and the gradual development of senescence-related phenotypes. However, it must be emphasized that direct experimental or clinical evidence linking these processes to endothelial senescence in the vertebral artery remains lacking. Therefore, the framework proposed here should be regarded as a biologically plausible but yet unvalidated integrative model. Its primary value lies in providing a conceptual basis for understanding the potential interplay among cervical degeneration, vertebral artery hemodynamic alterations, and endothelial dysfunction, rather than establishing a definitive causal relationship (Figure 3).

## 5 Endothelial senescence and vulnerability of the posterior circulation

Within this framework, the posterior circulation represents one of the vascular territories most sensitive to alterations in endothelial function because of its distinctive anatomical configuration and mechanisms of flow regulation (Zhang et al., 2022). Regulation of perfusion within the vertebrobasilar system relies heavily on endothelial-mediated dynamic responses, rendering it particularly vulnerable to reductions in vascular functional reserve (Maitas et al., 2023; Ota and Komiyama, 2022).The vertebrobasilar circulation supplies critical structures including the brainstem, cerebellum, and occipital lobes. Blood flow within this system depends on a relatively limited number of major arterial trunks and is maintained through finely tuned regulatory mechanisms that possess comparatively modest redundancy (Wake-Buck et al., 2012; Chen et al., 2015). Compared with the anterior circulation, the posterior circulation therefore has less compensatory capacity when confronted with fluctuations in blood flow, making it more sensitive to subtle changes in vascular functional status.

Within this physiological context, the vascular endothelium plays a central role in maintaining perfusion stability. Endothelial cells not only regulate vascular tone and blood flow distribution but also integrate mechanical, metabolic, and inflammatory signals to determine the adaptive range of vascular responses. As endothelial cells gradually acquire senescence-associated phenotypes, functional changes rarely manifest as abnormalities in a single measurable parameter. Instead, they are characterized by a progressive narrowing of the dynamic regulatory range through which vessels respond to environmental stimuli. In the posterior circulation, this reduction in regulatory reserve is more readily translated into functional instability (Diaz-Otero et al., 2016).

One important consequence of endothelial senescence is that vascular regulation shifts from a highly adaptable process toward a more rigid and passive mode of response (Seals et al., 2011). Under these conditions, vessels are not necessarily locked in a state of persistent constriction or dilation. Rather, they lose the capacity to adjust rapidly and effectively when perfusion demands change. As a result, even transient or modest fluctuations in blood flow may more easily translate into tissue-level hypoperfusion. For the posterior circulation, where compensatory mechanisms are inherently limited, this change substantially lowers the threshold for intolerance to physiological hemodynamic variation (Ungvari et al., 2025).

At the clinical level, this state of “reduced functional reserve” in the absence of persistent structural abnormalities is reflected in several vertebrobasilar disorders. For example, in Bow Hunter’s syndrome and rotational vertebrobasilar insufficiency, symptoms are often closely related to specific neck positions or movements (Regenhardt et al., 2022; Vilela et al., 2005). However, clinical observations suggest that these conditions cannot be fully explained by the degree of mechanical compression or the extent of blood flow reduction alone.

First, there is often a mismatch between imaging findings and clinical symptoms. Some patients develop obvious dizziness, visual disturbance, or brainstem-related symptoms despite only mild or transient changes in blood flow. In contrast, others may show clear anatomical compression but remain relatively stable or asymptomatic. Second, these symptoms are often fluctuating and reversible. In some patients, symptoms do not fully resolve even after returning to a neutral position (Wang et al., 2022b; Xue et al., 2023). This suggests that transient flow interruption alone cannot explain the full clinical picture. In addition, differences in individual tolerance to similar mechanical stimuli indicate that factors beyond anatomy are involved.

In this context, endothelial function may serve as a key link between mechanical stimuli and clinical manifestations. When endothelial cells enter a senescence-associated state, their ability to sense and rapidly respond to changes in blood flow is impaired. As a result, short-term fluctuations that would normally be buffered may instead lead to actual perfusion insufficiency. At the same time, low-grade inflammation and metabolic stress associated with endothelial senescence further reduce the ability of the vessel to adapt to repeated mechanical stimuli. Blood flow regulation shifts from a flexible, dynamic response to a more rigid and passive pattern.

From this perspective, mechanical compression or position-related flow changes can be viewed as triggering factors. In contrast, endothelial function largely determines the response threshold and recovery capacity of the vascular system. Therefore, even mild mechanical stress may produce clear symptoms in patients with reduced endothelial functional reserve. In individuals with preserved endothelial function, similar mechanical stimuli may be effectively compensated and remain clincally silent.

In addition, the inflammatory microenvironment and metabolic stress associated with endothelial senescence may further compromise the functional resilience of the posterior circulation (Li et al., 2025). Persistent low-grade inflammation and metabolic strain do not necessarily lead to overt structural vascular lesions. However, they can interfere with the coordinated signaling between endothelial cells and vascular smooth muscle cells, weakening the ability of vessels to adapt to postural changes, variations in physical activity, or systemic redistribution of blood flow (de la Riva et al., 2024). Clinically, this functional vulnerability often manifests as intermittent and nonspecific episodes of posterior circulation hypoperfusion, rather than as stable or predictable functional impairment.

It is also important to note that vulnerability of the posterior circulation frequently overlaps with age-related systemic changes (Jia et al., 2019). As vascular plasticity and reparative capacity decline with aging, the loss of regulatory capacity associated with endothelial senescence becomes increasingly difficult to compensate. Consequently, under similar external conditions, the posterior circulation may exhibit greater susceptibility to functional mismatch. This age-related vulnerability is therefore not determined solely by anatomical or hemodynamic factors but instead reflects the combined influence of endothelial state, microenvironmental alterations, and systemic vascular aging.

**FIGURE 4:**
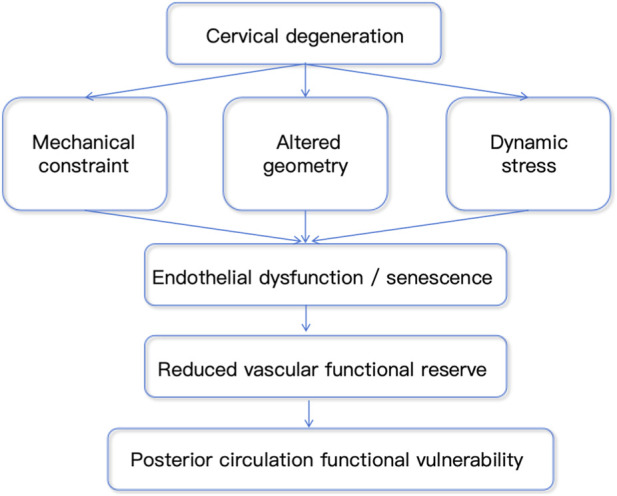
Conceptual model linking cervical degeneration, vertebral artery hemodynamics, and posterior circulation vulnerability. Cervical degeneration may influence vertebral artery function through multiple parallel pathways, including direct mechanical compression, alteration of vascular geometry, and motion-related dynamic stress. These factors can independently or synergistically contribute to hemodynamic instability, characterized by disturbed flow patterns, and reduced effective shear stress. The resulting environment may promote endothelial dysfunction and senescence, leading to a reduction in vascular functional reserve. In this framework, clinical manifestations are not determined solely by the degree of mechanical compression, but rather emerge from the integration of multiple biomechanical inputs and the functional state of the endothelium.

In summary, endothelial senescence itself does not directly equate to posterior circulation dysfunction, but it markedly compresses the functional reserve available for blood flow regulation, thereby lowering the tolerance of the posterior circulation to perfusion fluctuations. In many cases, this change does not present as persistent hemodynamic abnormalities but rather as a reduced capacity to adapt to normal physiological variation. In the setting of cervical degeneration, the vertebral artery is chronically exposed to spatial constraint, biomechanical stress, and microenvironmental alterations. These factors collectively push the posterior circulation to operate closer to the limits of its regulatory capacity. Under such circumstances, even in the absence of overt luminal stenosis, progressive endothelial functional decline may predispose the vertebrobasilar system to vascular dysregulation. Thus, posterior circulation disturbances associated with cervical degeneration may be better understood as the combined consequence of endothelial senescence and a specific anatomical–biomechanical context (Figure 4).

## 6 Therapeutic implications and future research directions

The preceding sections suggest that functional disturbances in this context do not arise from a single structural lesion. Rather, they reflect a gradual decline in vascular regulatory capacity under chronic stress. This view has important implications for therapy. Current approaches often focus on relieving structural compression or correcting transient hemodynamic changes. However, these strategies may not fully address the underlying problem. Greater emphasis should be placed on preserving endothelial function and maintaining stability of the vascular microenvironment. In this sense, shifting the focus from structural repair to functional resilience may better reflect the biology of the disease.

Within this framework, targeting endothelial senescence becomes particularly relevant. Strategies that reduce senescent cell burden, improve endothelial metabolism, or optimize the local biomechanical environment share a common goal. They aim to preserve endothelial function, rather than reverse established damage.This may be especially important in the posterior circulation, where compensatory capacity is limited. Even small improvements in endothelial function could help stabilize blood flow. Therefore, the key objective is to maintain functional reserve, rather than to target a single pathological pathway.

In terms of specific interventions, existing vascular biology studies provide several testable strategies.

First, approaches that reduce the burden of senescent endothelial cells have shown promise. Senolytic agents, such as dasatinib combined with quercetin or Bcl-2 family inhibitors, have been shown to reduce senescent cell burden and improve vascular function in aged animal models (Roos et al., 2016). Further studies indicate that these interventions can also decrease DNA damage and senescence-associated phenotypes in endothelial cells (Bloom et al., 2023b).Within the framework proposed in this study, if endothelial senescence in the vertebral artery is partly driven by chronic disturbed flow and low shear stress, reducing the senescent cell burden may improve the ability of endothelial cells to adapt to flow fluctuations. However, it should be noted that current evidence is mainly derived from large-vessel systems such as the aorta. Its relevance to the vertebral artery remains to be established.

Second, targeting metabolic stress also has a clear mechanistic basis. NAD^+^ precursor supplementation, such as NMN or NR, and activation of the SIRT1 pathway have been shown to improve endothelial metabolism, enhance mitochondrial function, and delay senescence-associated changes in vascular aging studies (Csiszar et al., 2019; Kiesworo et al., 2025). In endothelial cell models, restoration of NAD^+^ levels improves metabolic capacity and angiogenic potential (Harasim-Krawcewicz et al., 2026). Under conditions of chronic low perfusion and low shear stress, endothelial senescence may be partly driven by reduced metabolic reserve. In this context, restoring NAD^+^ levels may enhance the ability of endothelial cells to buffer transient flow changes. However, these findings are based mainly on general vascular models or *in vitro* systems. Direct evidence in the vertebral artery is still lacking.

Third, interventions targeting the hemodynamic environment itself may have specific value. For example, cervical posture correction, targeted exercise, and avoidance of extreme neck rotation may help modify the shear stress profile experienced by the vertebral artery. Dynamic studies have shown that head and neck position can significantly alter vertebral artery flow velocity and direction (Mahmoud et al., 2020; Kranenburg et al., 2019). These findings suggest that posture and movement can influence local hemodynamic conditions. In theory, increasing exposure to physiological laminar shear stress may help maintain protective pathways such as KLF2 and KLF4. However, there is currently no direct evidence that such interventions can modify endothelial senescence in the vertebral artery. This hypothesis requires further validation using combined hemodynamic and endothelial function assessments.

In patients with clear anatomical abnormalities or dynamic compression, surgical decompression or structural correction remains an important option. Previous studies have shown that these procedures can relieve vascular compression and improve blood flow as well as clinical symptoms in conditions such as Bow Hunter’s syndrome (Nguyen et al., 2015; Elizondo-Ramirez et al., 2024).From the perspective of this study, the potential benefit of these interventions may extend beyond mechanical decompression. They may also restore a more stable hemodynamic environment and indirectly improve endothelial function. However, current studies focus mainly on flow restoration and symptom relief. Data on endothelial function or senescence-related markers are limited. Future studies should include longitudinal assessment of both hemodynamic parameters and endothelial function to clarify these effects.

It needs to be emphasized that current experimental models often fail to fully capture the long-term and multifactorial stress environment experienced by the vertebral artery *in vivo*. Static culture conditions or models based on isolated mechanical or metabolic stimuli tend to underestimate the temporal heterogeneity and cumulative effects of multiple stressors encountered by endothelial cells in physiological settings. The above-mentioned strategies are not established treatment methods for vertebral artery endothelial aging, but rather hypothetical intervention pathways derived based on existing vascular biological evidence and the biomechanical framework proposed in this study. Its core value lies in proposing directions that can be further verified by experiments and clinical research, rather than being directly used to guide current clinical practice. Future studies should therefore aim to develop experimental systems that more closely reproduce physiological conditions, integrating dynamic mechanical forces, fluctuations in perfusion, and changes in metabolic state within a unified framework. Such approaches would allow a more accurate characterization of the initiation and progression of endothelial senescence.

In addition, the identification and validation of endothelium-specific senescence biomarkers will be critical for advancing this field. At present, assessment of vascular aging largely relies on indirect or nonspecific indicators that do not adequately reflect the continuous changes in endothelial functional status. Longitudinal and prospective research designs that track endothelial state over time may help clarify the relationship between endothelial aging and vascular functional decline and may further distinguish reversible functional fluctuations from progressively stabilized senescent phenotypes.

Overall, within the context of cervical degeneration, a chronic and largely irreversible structural and biomechanical background, vascular alterations of the vertebral artery may be better understood as a gradual imbalance in functional adaptation, rather than as the direct consequence of a single structural abnormality. From this perspective, endothelial senescence should not be viewed as a secondary phenomenon but rather as a central biological link connecting cervical degeneration, alterations in blood flow environment, and the vulnerability of posterior circulation function.
